# Remote Monitoring in Chronic Heart Failure Patients: Is Non-Invasive Remote Monitoring the Way to Go?

**DOI:** 10.3390/s21030887

**Published:** 2021-01-28

**Authors:** Jesse F. Veenis, Sumant P. Radhoe, Petra Hooijmans, Jasper J. Brugts

**Affiliations:** Erasmus MC, University Medical Center Rotterdam, Thorax Center, Department of Cardiology, 3000 Rotterdam, The Netherlands; s.radhoe@erasmusmc.nl (S.P.R.); p.hooijmans@erasmusmc.nl (P.H.); j.brugts@erasmusmc.nl (J.J.B.)

**Keywords:** remote monitoring, non-invasive monitoring, telemonitoring, heart failure

## Abstract

Heart failure (HF) is a major health care issue, and the incidence of HF is only expected to grow further. Due to the frequent hospitalizations, HF places a major burden on the available hospital and healthcare resources. In the future, HF care should not only be organized solely at the clinical ward and outpatient clinics, but remote monitoring strategies are urgently needed to guide, monitor, and treat chronic HF patients remotely from their homes as well. The intuitiveness and relatively low costs of non-invasive remote monitoring tools make them an appealing and emerging concept for developing new medical apps and devices. The recent COVID-19 pandemic and the associated transition of patient care outside the hospital will boost the development of remote monitoring tools, and many strategies will be reinvented with modern tools. However, it is important to look carefully at the inconsistencies that have been reported in non-invasive remote monitoring effectiveness. With this review, we provide an up-to-date overview of the available evidence on non-invasive remote monitoring in chronic HF patients and provide future perspectives that may significantly benefit the broader group of HF patients.

## 1. Introduction

Worldwide, approximately 26 million patients are currently diagnosed with heart failure (HF), and this population is rapidly growing [1]. Several factors, including an increase in awareness, improved diagnostic techniques, improved survival of coronary artery disease, increase in the prevalence of HF-related comorbidities such as hypertension and diabetes mellitus, and an aging population, contributes to this growth [2]. HF management places a major burden on healthcare resources due to frequent hospitalizations and outpatient visits [3]. Additionally, chronic HF is associated with increased mortality and morbidity [4].

Timely detection of congestion due to HF can prevent HF-related hospitalization, reduce the overall burden on health care resources, and improve patient outcomes [5,6]. Remote monitoring could be a crucial tool for the early detection of deterioration of HF. Furthermore, remote monitoring could also be used to stratify which patients are at high risk for deterioration and need frequent follow-up or outpatient attention and those who are at low risk and require less regular follow-up. It has been shown that the uptake and titration of guideline-recommended medical HF therapy could be improved further [7,8,9,10]. Remote monitoring strategies can be used to aid clinicians in the up-titration of guideline-recommended medical HF therapy [11]. Over the last years, multiple monitoring strategies, such as non-invasive remote monitoring (consisting of structured telephone support or non-invasively monitoring of parameters including body weight, blood pressure, and heart rate), monitoring using cardiac implantable electronic devices (such as implantable cardioverter-defibrillator or cardiac resynchronization therapy devices) and invasive remote hemodynamic monitoring, have been proposed and tested [12,13,14]. Considering the surge in medical technology apps, which will be boosted by the recent COVID-19 pandemic, it is essential to have an updated overview of the available tools and their evidence. In this review, we will focus on non-invasive remote monitoring tools in HF patients. Studies investigating non-invasive remote monitoring strategies can be divided into studies that have compared usual care with (I) structured telephone support or (II) non-invasive telemonitoring. In the case of non-invasive telemonitoring, patients were instructed to measure specific parameters (such as body weight, heart rate, or blood pressure), which were automatically sent to their health care team. In 2015 a Cochrane meta-analysis assessing the effects of both non-invasive remote monitoring strategies in chronic HF patients had been updated [12]. This Cochrane review reported a significant reduction in all-cause mortality for both structured telephone support, as well as non-invasive telemonitoring (Risk Ratio (RR) 0.87 (0.77–0.98) and RR 0.80 (0.68–0.94), resp.) and a significant reduction in HF-related hospitalizations (RR 0.85 (0.77–0.93) and RR 0.71 (0.60–0.83), resp.). However, the effects were relatively small and not convincingly positive, with the vast majority of studies being negative. This is important when new apps and e-health tools are developed based on old principles. However, since then, several new studies have been published which have reported more positive results if a structured approach is used in specific populations. Therefore, this review aims to provide an overview of the currently available evidence on both non-invasive remote monitoring strategies of chronic HF patients.

## 2. Methods for Study Selection

We included randomized controlled trials as well as clinical studies comparing HF management delivered via structured telephone support or non-invasive home telemonitoring with usual post-discharge care for people with heart failure living within the community. We included only studies that have been published in full in the peer-reviewed literature. We excluded any studies that did not report data for any of our outcomes of interest in an extractable format (all-cause mortality, all-cause hospitalization, HF-related hospitalization, or quality of life) or those who used home visits or additional outpatient clinics. Additionally, all included studies reported data of only adult patients (aged 18 years or older) of either sex, any ethnic group, with a definitive diagnosis of HF. Patients could have been recently discharged from a cardiac clinic after an episode of decompensation or being recruited in a stable setting from outpatient clinics, as well as studies reporting data on general cardiac or chronic disorder rather than specifically HF. A combination of the following search terms were used: ‘heart failure’, ‘heart or cardiac or myocard’, and ‘failure or insufficiency or decompensation’, in combination with ‘telemedicine’, ‘telecommunication’, ‘telemonitoring’, ‘teleconsult’, ‘telehealth’, ‘home monitoring’, ‘home care’, ‘ambulatory monitoring’, ‘telehome’, ‘ehealth’ or ‘mobile health’.

We searched the MEDLINE, pubmed, database on 1 September 2020, and performed the following. All titles and abstracts were checked for relevance to the review topic by two authors, independently. In case of disagreement, a third author would check the article as well. All data relevant data were extracted from the articles.

## 3. Structured Telephone Support versus Usual Care

We identified 31 studies that compared structured telephone support with usual care, which included a total of 11,270 patients [15,16,17,18,19,20,21,22,23,24,25,26,27,28,29,30,31,32,33,34,35,36,37,38,39,40,41,42,43,44,45]. The study characteristics, as well as the reported outcomes on all-cause mortality, all-cause hospitalization, and HF-related hospitalization rates of the five largest studies, representing 49% (5560) of all patients, will be discussed in detail below [19,21,22,23,34]. The study characteristics and outcomes of the other 26 studies will be summarized below.

### 3.1. Chaudhry et al. (Tele-HF Trial)

In 2010, Chaudhry et al. published the results from the Telemonitoring to Improve Heart Failure Outcomes (Tele-HF) trial, including 1653 recently hospitalized chronic (e.g., unstable) HF patients with a median age of 61 years, 58% were men, and 57% were in the New York Heart Association (NYHA) class III or higher [23]. The prescribed background was relatively low, with 79% of patients receiving a beta-blocker, 67% a renin-angiotensin system (RAS)-inhibitor, and only 33% a mineralocorticoid receptor antagonist (MRA). The patients were followed for six months. During this period, no significant differences in the all-cause mortality (odds ratio (OR) 0.98 (0.75–1.28), all-cause hospitalization (OR 1.08 (0.89–1.31)) or HF-related hospitalization rates (OR 1.04 (0.84–1.30)) were observed. Overall, a marginal, non-significant reduction in all-cause mortality and both all-cause and HF-related hospitalizations occurred more often in HF patients receiving structured telephone support.

### 3.2. Ferrante et al. (DIAL Trial)

The results from the Randomized Trial of Phone Intervention in Chronic Heart Failure (DIAL) were published in 2010 by Ferrante et al. [22]. They included 1518 stable chronic HF patients, with a mean age of 65 years, 71% were men, and 49% were in an NYHA class III or IV. The prescribed background was relatively low; only 62% received a beta-blocker, 93% a RAS-inhibitor, and 32% an MRA. All patients were followed for 12 months; during this period, no significant reduction in all-cause mortality (OR 0.95 (0.75–1.20)) or all-cause hospitalizations (OR 0.82 (0.66–1.01)) were observed. However, a significant reduction in the number of HF-related hospitalizations was reported (OR 0.71 (0.55–0.91)) in patients receiving structured telephone support.

### 3.3. Galbreath et al.

Galbreath et al. included 1069 stable chronic HF patients, with a mean age of 71 years, 71% were men, and 24% were in an NYHA class III or IV and reported their results in 2004 [34]. The background therapy was not frequently prescribed, with 47% of patients receiving a beta-blocker and 73% receiving a RAS-inhibitor. No data on MRA prescription rates were reported. The follow-up duration was 18 months, and during this period, no significant difference in the all-cause mortality rates (OR 0.70 (0.47–1.04)) was reported. The study did not report data on all-cause or HF-related hospitalization rates.

### 3.4. Angermann et al. (INH Study)

The results from the Interdisciplinary Network for Heart Failure (INH) study performed by Angermann et al. was published in 2012 [19]. A total of 715 unstable HF patients (mean age 69 years, 71% males and 40% in an NYHA class III or higher) were included. These patients frequently received background HF therapy; 80% received a beta-blocker, 88% a RAS-inhibitor, and 42% an MRA. A significant reduction in the all-cause mortality rates (OR 0.63 (0.42–0.96)) was observed during the six month follow-up period in patients receiving structured telephone support. No significant differences were observed in the all-cause or HF-related hospitalization rates (OR 1.14 (0.84–1.57) and OR 0.79 (0.49–1.25), respectively).

### 3.5. Baker et al.

In 2011, Baker et al. published the results of their study, including 605 stable chronic HF patients (mean age 61 years, 52% were men, and 31% were in an NYHA class III/IV) [21]. Many patients received HF background therapy; a beta-blocker was prescribed in 81%, RAS-inhibitor in 82%, and MRAs in 27%. The patients were followed for one month, and during this period, no significant difference in the all-cause mortality was observed (OR 0.20 (0.01–4.13)) between HF patients receiving usual care or structured telephone support. The study did not report data on all-cause or HF-related hospitalization rates.

### 3.6. Other Studies

A summary of the study characteristics for the 26 other structured telephone support studies is shown in Table 1. As shown, large differences in the sample sizes and patient demographics exist between the studies. Between 20 to 462 patients were included in these studies, with a mean age ranging from 57 to 76 years. The follow-up duration ranged from three to 12 months. Additionally, significant differences in the reported background therapy were reported. Between 4% to 87% of the patients with structured telephone support received a beta-blocker, 54% to 95% received a renin-angiotensin system (RAS) inhibitors, and 6% to 63% received a mineralocorticoid receptor antagonist (MRA).

**Figure 1:**
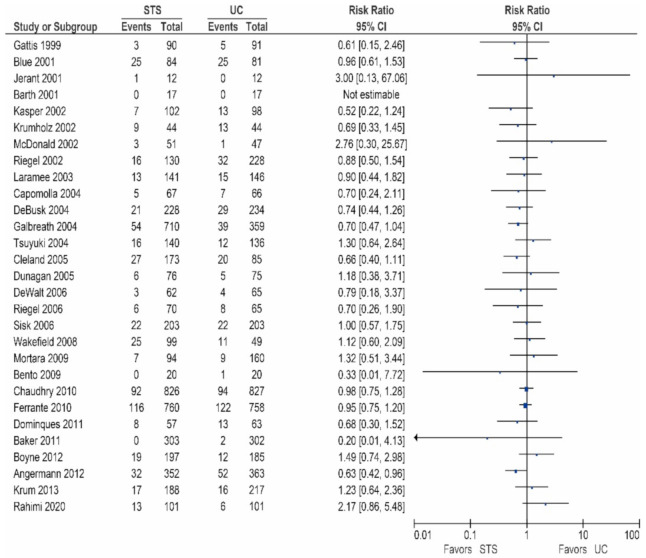
All-cause mortality in patients receiving structured telephone support versus usual care.

Of the 26 other studies, 24 studies reported data on the all-cause mortality, these results are shown in Table 2/Figure 1 [15,17,18,20,24,25,27,28,29,30,31,32,33,35,36,37,38,39,40,41,42,43,44,45]. As shown, 13 studies did report a non-significant reduction in all-cause mortality [20,25,29,30,32,35,36,37,38,40,41,43,45], while 11 studies did not show a reduction in the all-cause mortality [15,17,18,24,27,28,31,33,39,42,44].

**Figure 2:**
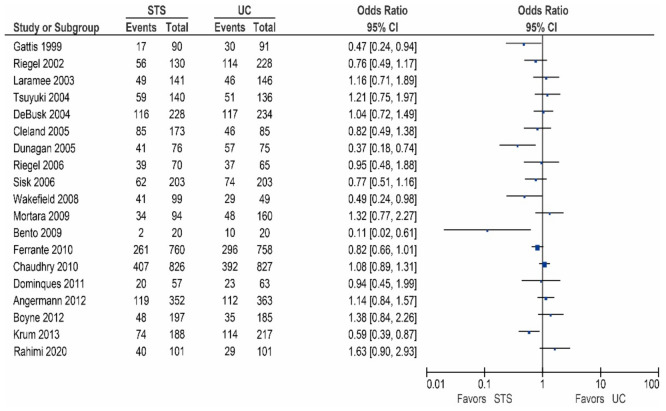
All-cause hospitalization in patients receiving structured telephone support versus usual care.

Sixteen of the other studies reported data on all-cause hospitalization rates, as shown in Table 2/Figure 2 [15,17,18,20,24,25,27,28,29,31,32,33,35,37,38,45]. Of these studies, five demonstrated a significant reduction in the all-cause hospitalization rates [17,25,27,31,45], while five studies reported a non-significant reduction [20,28,29,32,38]. In contrast, six studies did not report a beneficial effect [15,18,24,33,35,37].

**Figure 3:**
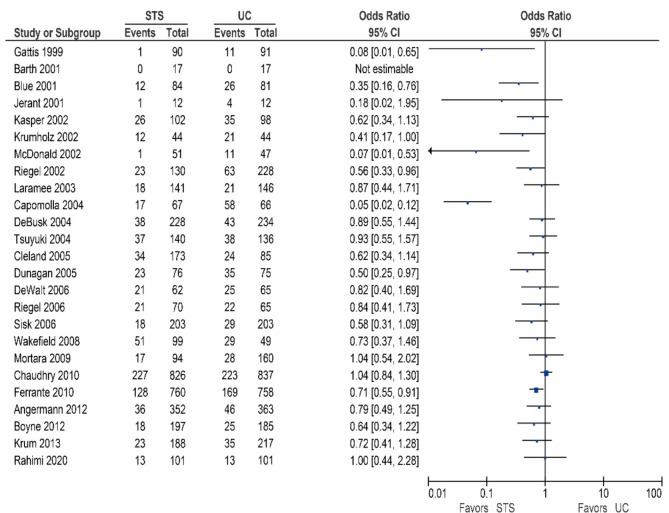
Heart Failure (HF)-related hospitalizations in patients receiving structured telephone support versus usual care.

Twenty-two of the 26 other studies reported data on the HF-related hospitalization rates, and are shown in Table 3/Figure 3 [15,17,18,24,27,28,29,30,31,32,33,35,36,37,38,39,40,41,42,43,44,45]. Of these studies, six reported a significant reduction in the HF-related hospitalization rates [31,36,38,39,42,45], while 13 studies showed a non-significant reduction [17,18,27,28,29,30,32,33,35,37,40,41,42]. In contrast, three studies reported no beneficial effect [15,24,44].

### 3.7. Quality of Life, Symptoms, and Functional Performance

A variety of questionnaires, including Short Form 12 Item (SF-12), Short Form 36 Item (SF-36), Minnesota Living with Heart Failure Questionnaire (MLWHFQ), EuroQol five-dimension scale (EQ-5D), Patient Health Questionnaire-9 (PHQ-9), and other tools were used to evaluate the quality of life.

Thirteen studies have assessed the effects of structured telephone support on the quality of life. The quality of life of patients who received structured telephone support improved significantly more than standard care in seven studies [19,21,22,26,28,41,45], and non-significantly in one study [27]. In contrast, five studies did not show a larger improvement in the quality of life [15,16,29,30,46].

In total, three of the five studies investigating structure telephone support reported a significantly bigger improvement of symptoms compared to the usual care [19,34,41], while two studies did not find a difference [15,32].

Of the two studies reporting functional performance data in patients receiving structured telephone support, one study demonstrated a bigger improvement in the intervention group [16], while the other study did not observe a difference [34].

## 4. Non-Invasive Telemonitoring versus Usual Care

In total, 30 studies investigating non-invasive telemonitoring have been identified, including a total of 8892 patients [24,32,42,47,48,49,50,51,52,53,54,55,56,57,58,59,60,61,62,63,64,65,66,67,68,69,70,71,72,73]. The study characteristics, as well as the reported outcomes on all-cause mortality, all-cause hospitalization, and HF-related hospitalization rates of the five largest studies, representing 52% (4606) of all patients, will be discussed in detail below [24,51,52,61,64]. The study characteristics and outcomes of the other 25 studies will be summarized below [32,42,47,48,49,50,53,54,55,56,57,58,59,60,62,63,65,66,67,68,69,70,71,72,73].

### 4.1. Koehler et al. (TIM-HF2 Trial)

The results from the Telemedial Interventional Management in Heart Failure II (TIM-HF2) trial have been published in 2018 by Koehler et al. [51]. In this study, 1571 stable chronic HF patients (mean age 70 years, 70% was men and 48% were in an NYHA class III/IV) were randomized towards usual care or remote telemonitoring consisting of daily transfers of body weight, blood pressure, heart rate, heart rhythm, peripheral capillary oxygen saturation, and self-rated health status. Many included patients received HF background therapy; 92% received a beta-blocker, 83% a RAS-inhibitor, and 55% an MRA. The patients were followed for 12 months, and the compliance with the daily data transfer was 95% in the intervention patient group. During this period, a significant reduction in all-cause mortality (OR 0.64 (0.45–0.90) was observed in HF patients receiving non-invasive telemonitoring, while no beneficial effect on the all-cause hospitalization rates (OR 1.04 (0.84–1.29) was reported. No individual data on HF-related hospitalization were reported.

### 4.2. Ong et al. (BEAT-HF Trial)

In 2016 Ong et al. published the Better Effectiveness After Transition-Heart Failure (BEAT-HF) trial [52]. This study included 1437 hospitalized HF patients (median age 74 years, 54% were men and 75% was in an NYHA class III or higher) and randomized them towards usual care or remote telemonitoring, consisting of daily data transfers of blood pressure, heart rate, symptoms, and body weight in addition to health coaching telephone calls and usual care. The use of HF background therapy was relatively low; beta-blockers were prescribed to 75% of all patients, RAS-inhibitors to 56%, and only 19% received an MRA. The patients were followed for up to six months. During this period, the telemonitoring adherence was 51.7% in the patients who were remotely monitored. A non-significant reduction in all-cause mortality (OR 0.86 (0.64–1.16)) was reported, while no beneficial effect on the all-cause hospitalization rates (1.07 (0.87–1.31)) was shown in chronic HF patients receiving non-invasive telemonitoring. Data on HF-related hospitalization rates were not reported.

### 4.3. Koehler et al. (TIM-HF Study)

The Telemedical Interventional Monitoring in Heart Failure (TIM-HF) trial by Koehler et al., published in 2011, included 710 stable chronic HF patients (mean age 67 years, 81% was male and 50% were in NYHA class III or IV) [61]. These patients were randomized towards usual care or remote telemonitoring, consisting of electrocardiogram (ECG), blood pressure, and body weight measurements on top of usual care. Relative a high percentage of patients received HF background therapy; 93% received a beta-blocker, 95% a RAS-inhibitor, and 64% an MRA. The median follow-up was 26 months. During this period, 81% of all patients receiving telemonitoring performed at least 70% of all daily data transfers. During this period no effects on the all-cause mortality (OR 0.99 (0.65–1.48), all-cause hospitalization (OR 1.17 (0.87–1.57) or HF-related hospitalization rates (OR 0.84 (0.58–1.22)) were observed between HF patients receiving non-invasive telemonitoring or usual care.

### 4.4. Mortara et al. (HHH Study)

Mortara et al. published the results from the Home of Hospital in Heart Failure (HHH) in 2009 [24]. In total 461 stable chronic HF patients (mean age 60 years, 85% were men and 40% was in NYHA class III/IV) were included and randomized towards usual care or telemonitoring, consisting of blood pressure, body weight, heart rate, and signs and symptoms measurements on top of usual care. Many patients received HF background therapy, with 87% of patients receiving a beta-blocker and RAS-inhibitor. Patients were followed for 12 months, during this period no reduction in all-cause mortality (OR 1.44 (0.54–3.87)), all-cause hospitalizations (OR 1.24 (0.73–2.10)) or HF-related hospitalizations (OR 1.02 (0.53–1.96)) were observed in patients receiving non-invasive telemonitoring.

### 4.5. Giordano et al.

In 2009, Giordano et al. published the results from their study, including 460 unstable chronic HF patients (mean age 57 years, 85% were men, and 40% was in an NYHA class III or higher) [64]. The use of background HF therapy was relatively low; 72% of patients received a beta-blocker, 94% a RAS-inhibitor, and 62% an MRA. The patients randomized towards telemonitoring received regular remote monitoring using ECG on top of usual care. The follow-up period was 12 months, during which a significant reduction in the all-cause mortality (OR 0.39 (0.18–0.82)), all-cause hospitalization (OR 0.57 (0.39–0.84)), and HF-related hospitalization rates (OR 0.49 (0.32–0.76)) were observed in chronic HF patients receiving non-invasive telemonitoring compared to patients receiving usual care.

### 4.6. Other Studies

A summary of the study characteristics for the 25 other structured telephone support studies is shown in Table 3. As shown, large differences in the sample sizes and patient demographics exist between the studies. Between 20 to 426 patients were included in these studies, with a mean age ranging from 54 to 82 years. The follow-up duration ranged from one to 48 months. Additionally, significant differences in the reported background therapy were reported. Between 38% to 98% of the patients with structured telephone support received a beta-blocker, 66% to 100% received a renin-angiotensin system (RAS) inhibitors, and 21% to 58% received a mineralocorticoid receptor antagonist (MRA).

Twelve of these other studies reported data on monitoring adherence, ranging from 46% up to 95% [47,48,50,54,55,56,59,60,63,65,69,73]. Importantly to note, the monitoring strategy’s adherence decreased when the patients had to perform multiple measurements [48,50,73]. Additionally, the adherence decreased over time [48,50,55,56]. Surprisingly, the adherence also decreased in the weeks after hospitalization [47]. These results highlight that some studies’ adherence was far from optimal and could be even lower in a ‘real world’ setting. Treating clinicians should reinforce the importance of adherence to the monitoring strategies by the patients to optimize the effects of non-invasive remote monitoring strategies.

**Figure 4:**
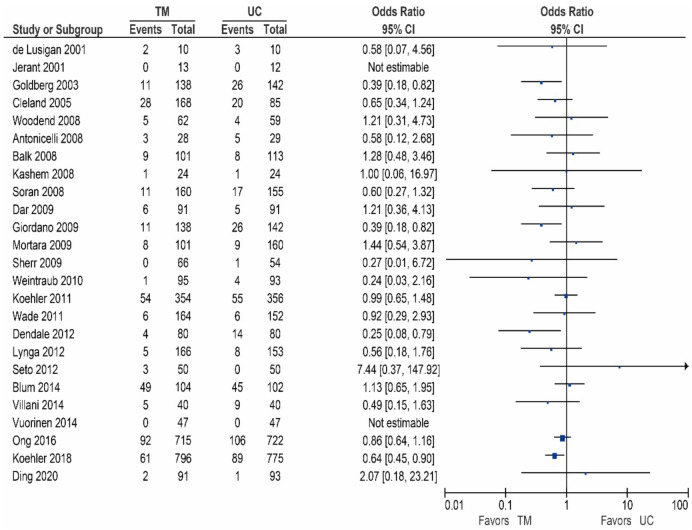
All-cause mortality in patients receiving non-invasive telemonitroing versus usual care.

Of the 25 other studies, 20 studies reported data on the all-cause mortality, these results are shown in Table 4/Figure 4 [32,42,48,53,54,55,56,58,59,60,62,63,65,66,67,68,69,70,71,73]. As shown, two studies reported a significant reduction [59,71], ten studies did report a non-significant reduction in all-cause mortality [32,54,55,58,60,62,63,67,70,73], while eight studies did not show a reduction in the all-cause mortality [42,48,53,56,65,66,68,69].

**Figure 5:**
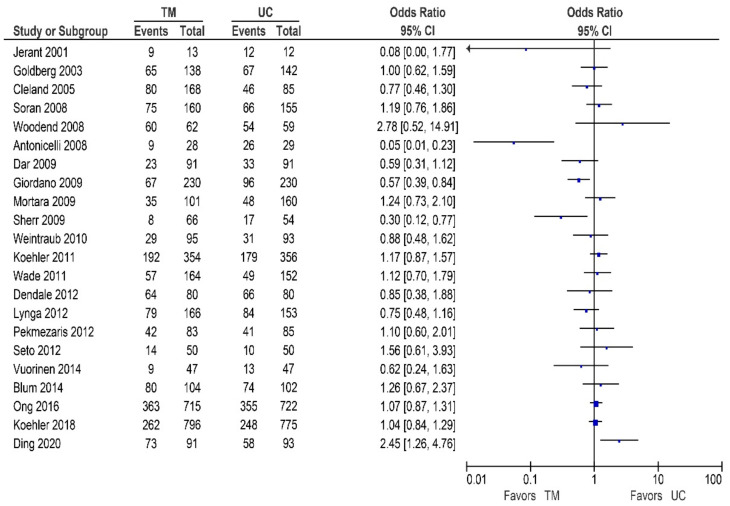
All-cause hospitalization in patients receiving non-invasive telemonitoring versus usual care.

Seventeen of the other studies reported data on all-cause hospitalization rates, as shown in Table 4/Figure 5 [32,42,48,53,55,56,57,58,59,60,62,65,66,67,70,71]. Of these studies, two demonstrated a significant reduction in the all-cause hospitalization rates [63,70], while seven studies reported a non-significant reduction [32,42,53,58,59,62,65]. In contrast, eight studies did not report a beneficial effect [48,55,56,57,60,66,70,71].

**Figure 6:**
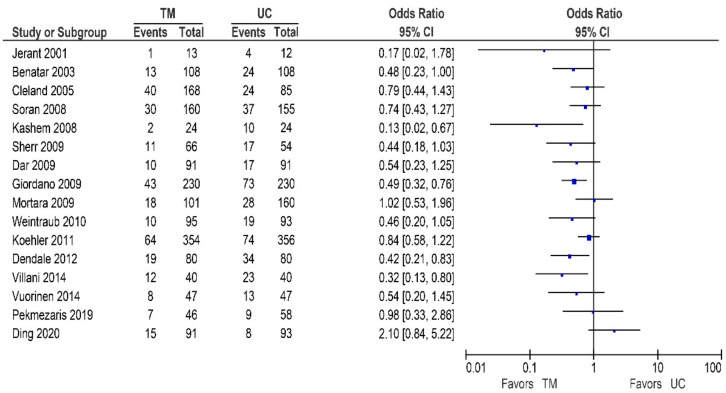
HF-related hospitalizations in patients receiving non-invasive telemonitoring versus usual care.

Thirteen of the 25 other studies reported data on the HF-related hospitalization rates, and are shown in Table 4/Figure 6 [32,42,48,53,54,57,59,62,63,65,67,68,72]. Of these studies, three reported a significant reduction in the HF-related hospitalization rates [54,59,68], while nine studies showed a non-significant reduction [32,42,53,57,62,63,65,67,72]. In contrast, only one study reported no beneficial effect [48].

### 4.7. Quality of Life, Symptoms, and Functional Performance

Seventeen studies investigating non-invasive telemonitoring reported data on the quality of life. Seven of these studies reported a significantly larger improvement in the quality of life compared to the standard care [48,52,54,56,61,67,70], while four studies demonstrated a non-significant difference [60,71,72,73]. In total, six studies did not demonstrate a beneficial effect on the quality of life [49,51,55,65,66,69].

Two of the five studies including telemonitoring strategies reported a significantly larger improvement of symptoms compared to the standard care [54,63], while three studies did not demonstrate a beneficial effect of non-invasive telemonitoring [32,56,61].

Only one study investigated the effect of non-invasive telemonitoring on functional performance and did not found a beneficial effect of this intervention [48].

## 5. Overview of Available Studies for Non-Invasive Remote Monitoring in Heart Failure Management: Clinical Interpretations

As shown above, considerable heterogeneity has been observed in the reported results of all-cause mortality, all-cause hospitalization, HF-related hospitalization, and quality of life for both structured telephone support and non-invasive telemonitoring. Considering all the published results, the following overall effects could be observed.

### 5.1. Structured Telephone Support

Based on all the published results, structured telephone support appears to have a small beneficial effect on all-cause mortality and all-cause hospitalization rates, although it might not be significant. In contrast, a more clear beneficial impact on the HF-related hospitalization rates has been observed. Additionally, this remote monitoring strategy could improve the quality of life. However, large heterogeneity has been observed between the published studies. Several reasons might explain the observed differences. Firstly, the number of included patients and the study design different significantly between the studies. Additionally, we observed a clear association between the year of publication and the treatment effect, with older studies showing more often a beneficial effect. Finally, the heterogeneity might be explained by differences in the follow-up period. Studies using a shorter follow-up period were more likely to demonstrate a beneficial effect. The reasons for the inconsistency in the reported results are discussed in more detail down below.

### 5.2. Non-Invasive Telemonitoring

Overall, non-invasive telemonitoring strategies might significantly reduce all-cause mortality and HF-related hospitalization rates. In contrast, no significant reduction in the all-cause hospitalization rates has been observed. The quality of life, symptom burden, and functional performance improved in patients who received non-invasive telemonitoring. However, significant heterogeneity has been observed in the published results. Studies demonstrating a beneficial effect were more likely to include patients who were recently hospitalized for HF, representing patients with unstable HF. Additionally, studies including a more extensive remote monitoring strategy, using multiple parameters, more often reported a beneficial effect. Additionally, more recent published studies less often demonstrated a beneficial effect, compared to older studies. These reasons for inconsistency in the reported results are discussed in more detail down below.

## 6. Reasons for Inconsistent Results

As demonstrated, large heterogeneity in the reported effects of non-invasive remote monitoring strategies on all-cause mortality, all-cause hospitalization, and HF-related hospitalizations exists. Several differences in study design and patient characteristics might explain the inconsistency in the reported results. So was the percentage of studies that included patients who were hospitalized due to HF (e.g., unstable HF patients) lower in studies who did not report a beneficial effect of structured telephone support or non-invasive remote monitoring on the all-cause mortality, all-cause hospitalization, or HF-related hospitalization rates. Hospitalization for HF is associated with an increased risk for mortality as well as rehospitalizations [5,74]. In these unstable HF patients, non-invasive remote monitoring has the largest potential to improve their clinical outcomes and reduce the HF-related hospitalization rates. In contrast, stable HF patients might already be in an ideal clinical condition, and adding non-invasive remote monitoring would not lead to further optimization of their condition.

It has been suggested that the more recently published non-invasive remote monitoring studies would have a reduced benefit in preventing all-cause mortality, all-cause hospitalization, and HF-related hospitalization rates [12]. We showed similar results, with most studies that showed no beneficial effects of non-invasive remote monitoring were published in 2008 or more recent. Over the last decades, cardiac imaging, diagnostic testing, pharmacological treatment, and device therapy have evolved continuously [75]. Results from earlier perform studies might not reflect the current state of HF care, as indicated by the lower uptake of the guideline-recommended background therapy in the earlier published studies. This could have significantly impacted the results, with less optimized patients included in the earlier studies and more optimized patients in the later studies.

Additionally, the follow-up duration in the studies ranged from one month up to four years. We observed that most studies that demonstrated a reduction of all-cause mortality had a follow-up period of six months or shorter. Studies with a follow-up period longer than six months reported more often no difference in the all-cause mortality. Similar results were found in the most recent Cochrane review [12]. These results indicate that non-invasive remote monitoring might improve the clinical outcomes in the short term, but that long term survival remains unaffected. Structured telephone support studies with a follow-up period of six months or shorter reported more often a beneficial effect on the hospitalization rates, while studies with a longer follow-up demonstrated more often no reduction.

In contrast, such difference was not found in studies analyzing non-invasive remote telemonitoring, as also have been demonstrated by the most recent Cochrane meta-analysis [12]. Many structured telephone support monitoring strategies focused on patient education. This could help maintain an optimal clinical state during the short term but would be ineffective in detecting an upcoming deterioration of HF in time. Especially since the interval of the telephone calls was only once every two weeks or even less often. In contrast, patients receiving non-invasive remote telemonitoring were instructed to take daily readings. Therefore, in these patients, signals of imminent HF deterioration could be detected in time, and hospitalization might be prevented, even during a longer follow-up period.

Finally, the variables included in the non-invasive remote telemonitoring studies varied widely. This limits the comparability of the studies and might explain some of the observed inconsistent results. Furthermore, in some studies, adherence to the telemonitoring strategy declined rapidly, reducing these monitoring strategies’ effectiveness. Improving patient participation in the monitoring strategies, ensuring that the patient performs the monitoring readings daily regardless of their situation, is crucial for developing an effective remote monitoring strategy.

## 7. Future Perspectives

Currently, the 2016 European Society of Cardiology (ESC) Guidelines for the diagnosis and treatment of acute and chronic heart failure does not provide any recommendation for the use of non-invasive remote monitoring [76]. In contrast, the use of invasive remote monitoring of pulmonary artery pressures or multiparameter monitoring based on implantable cardioverter-defibrillator may be considered (Class IIb recommendation) [76]. In contrast, the 2013 American College of Cardiology (ACC) and American Heart Association (AHA) Guideline for the management of heart failure recommended using effective systems to coordinate HF care to provide the guideline-recommended medical therapy and prevent hospitalizations (class I recommendation), although remote monitoring is not explicitly recommended [77]. However, in the most recent updated ACC/AHA guideline of 2017, no new recommendation on remote monitoring has been included [78]. Since more evidence shows a beneficial effect of remote monitoring and provides incremental information that could be used in the titration of HF treatment, we expect that in the newest guidelines of both the ESC and ACC/AHA, remote monitoring of chronic HF patients will receive a more specific positive recommendation. Since the more recent published studies on non-invasive remote monitoring strategies have shown less often a beneficial effect on preventing HF-related hospitalizations, this strategy might be more indicated to be used in less symptomatic HF patients to guide the titration of their HF therapy. In contrast, more invasive remote monitoring strategies could be recommended in more symptomatic HF patients.

The additional information, provided by non-invasive remote monitoring strategies, can be used to prevent HF decompensations, and alert treating clinicians for an imminent HF-related hospitalization. Currently, most studies investigating non-invasive remote monitoring have focused on this aspect. However, these strategies are limited due to their reactive design, leaving only a short period for the treating clinicians to react and prevent HF-related hospitalizations [6]. Instead, an active strategy could be used as well. Non-invasive remote monitoring could also be used to assess an ideal target for each patient. The provided feedback could guide the medical therapy to reach and maintain the patients as close to this target as possible, improving their clinical status, keeping them as stable as possible, and potentially allowing for cardiac remodeling and improving their survival [79]. Both a reactive and active strategy should not exclude each other and should be incorporated into one remote monitoring system. Reacting to imminent HF deterioration should be ideally coordinated from a central national center, allowing for timely intervention. In contrast, the active strategy, consisting of optimizing the HF therapy, should be managed by the local health care teams, who are in close contact with the patients [80].

Furthermore, the efficacy of newly introduced HF drugs, as well as invasive procedures (such as valvular interventions) could be monitored and analyzed using remote monitoring strategies. The feedback provided by these remote monitoring strategies can be used to better understand the (lack of) effects of these new treatment options. Additionally, the effects of treatment changes can be used to determine whether more invasive interventions are indicated.

Recently, studies investigating invasive remote monitoring strategies have shown a beneficial effect in more symptomatic patients [81,82,83,84]. However, these invasive strategies are limited due to their higher costs and its invasive nature. We believe that these invasive strategies should only be used in more symptomatic and more ill patients. Non-invasive remote monitoring strategies are easier to be widely implemented and should be used to monitor less symptomatic chronic HF patients.

In the future, the results from studies investigating remote monitoring in chronic HF patients should be analyzed by a trans-disciplinary team. Thereby, new technologies, such as artificial intelligence and machine learning, could be used to determine effective remote monitoring strategies and could highlight new inroads for further studies.

## 8. Conclusions

Despite some inconsistency in the reported results on the effectiveness of non-invasive remote monitoring in chronic HF patients, the overall combined results demonstrated a small beneficial effect on the overall survival, HF-related hospitalizations, and adherence to the guideline-recommended pharmacological therapy. Due to its simplicity, non-invasive nature, and relatively low costs, non-invasive remote monitoring is desirable and to be recommended in lower risk or less symptomatic chronic HF patients. As the volume of HF patients is very high, the impact of non-invasive remote monitoring strategies could have a large impact at not too high costs. More symptomatic and complex higher risk HF patients would likely benefit more from invasive remote monitoring strategies, but at a higher cost.

## Figures and Tables

**Table 1:** Baseline characteristics and background HF therapy in studies investigating structured telephone support in HF patients.

Author (Year)	Study Acronym	Number of Patients	Age	Male (%)	NYHA III/IV (%)	Duration of Follow-Up	Background HF Therapy		
							Beta-Blockers	RAS-Inhibitors	MRAs
Rahimi et al. (2020) [15]	SUPPORT-HF 2	202	71.3 ± 11.1	71.3	40.5	6 months	NA	NA	NA
Gingele et al. (2019) [16]	TEHAF	382	71.4 ± 11.2	59.2	42.7	12 months	82.0	89.9	NA
Krum et al. (2013) [17]	CHAT	405	73.0 ± 10.5	63.1	41.4	12 months	61.4	84.2	26.1
Boyne et al. (2012) [18]	TEHAF	382	71.4 ± 11.2	59.2	42.7	12 months	81.1	89.0	NA
Angermann et al. (2012) [19]	INH	715	68.6 ± 12.2	70.6	39.9	6 months	79.9	88.1	41.8
Domingues et al. (2011) [20]		111	63 ± 13	57.7	97.3	3 months	NA	NA	NA
Baker et al. (2011) [21]		605	60.7 ± 13.1	51.9	30.9	1 month	81.3	82.1	27.4
Ferrante et al. (2010) [22]	DIAL	1518	65.0 ± 13.3	70.8	49.4	12 months	61.6	92.9	32.2
Chaudhry et al. (2010) [23]	Tele-HF	1653	61 (51–73)	58.0	57.3	6 months	79.2	66.9	32.8
Mortara et al. (2009) [24]	HHH	461	60 ± 12	85.0	39.9	12 months	87	87	NA
Bento and Brofman (2009) [25]		40	57.5 ± 9.4	70.0	37.5	6 months	85.0	75.0	62.5
Brandon et al. (2009) [26]		20	60 (49–69)	45.0	25.0	3 months	NA	NA	NA
Wakefield et al. (2008) [27]		148	69.3 ± 9.6	98.6	71.6	12 months	NA	NA	NA
Sisk et al. (2006) [28]		406	59.4 ± 13.7	53.7	59.1	12 months	52.0	NA	NA
Riegel et al. (2006) [29]		134	72.1 ± 11.0	46.3	81.3	6 months	54.0	74.6	11.1
DeWalt et al. (2006) [30]		123	62.5 ± 10.0	49.2	50.1	12 months	63.3	73.3	NA
Dunagan et al. (2005) [31]		151	70.0 ± 13.3	43.7	80.1	12 months	NA	70.2	NA
Cleland et al. (2005) [32]	TEN-HMS	426	67.2 ± 11.6	77.2	34.0	8 months	80.9	81.0	49.1
Tsuyuki et al. (2004) [33]		276	71.5 ± 12	58.0	26.4	6 months	42.8	84.8	13.4
Galbreath et al. (2004) [34]		1069	70.9 ± 10.3	71	24	18 months	47	73	NA
DeBusk et al. (2004) [35]		462	72 ± 11	51.1	50.1	12 months	NA	NA	NA
Capomolla et al. (2004) [36]		133	57 ± 10	88.0	33.1	12 months	NA	84.2	21.1
Laramee et al. (2003) [37]		287	70.7 ± 11.8	54.4	35.9	3 months	NA	NA	NA
Riegel et al. (2002) [38]		358	73.9 ± 12.4	48.9	96.9	6 months	16.9	53.6	NA
McDonald et al. (2002) [39]		98	70.8 ± 10.5	66.3	2.3 ± 0.6	3 months	NA	61.2	NA
Krumholz et al. (2002) [40]		88	73.8 ± 9.5	56.8	NA	12 months	40.9	59.1	NA
Kasper et al. (2002) [41]		200	61.9 ± 14.3	60.5	58.5	6 months	39.0	94.5	NA
Jerant et al. (2001) [42]		37	70.1 ± 12.1	45.9	35.1	12 months	37.8	67.6	27.0
Blue et al. (2001) [43]		165	75.5 ± 8.3	57.6	77.0	12 months	4.2	71.5	5.5
Barth (2001) [44]		34	75.2 ± 8.4	47.1	NA	6 months	NA	NA	NA
Gattis et al. (1999) [45]	PHARMA	181	67.2 (55.0–74.5)	68.0	33.1	6 months	NA	77.9	NA

HF, heart failure; MRA, mineralocorticoid receptor antagonist; NA, not available; NYHA, New York Heart Association; RAS, renin-angiotensin system.

**Table 2:** Clinical outcomes of structured telephone support studies.

Author (Year)	Number of Patients		All-Cause Mortality			All-Cause Hospitalization			HF-Related Hospitalization			Quality of Life
			Number of Events		OR (95% CI)	Number of Events		OR (95% CI)	Number of Events		OR (95% CI)	
	STS	UC	STS	UC		STS	UC		STS	UC		
Rahimi et al. (2020) [15]	101	101	13	6	2.17 (0.86–5.48)	40	29	1.63 (0.90–2.93)	13	13	1.00 (0.44–2.28)	*MLWHFQ* STS −0.30; UC −0.66 *p* = 0.63
Gingele et al. (2019) [16]	197	185	NA	NA	NA	NA	NA	NA	NA	NA	NA	*EQ-5D* STS +0.01; UC +0.02 *p* = 0.83
Krum et al. (2013) [17]	188	217	17	16	1.23 (0.64–2.36)	74	114	0.59 (0.39–0.87)	23	35	0.72 (0.41–1.28)	NA
Boyne et al. (2012) [18]	197	185	19	12	1.49 (0.74–2.98)	48	35	1.38 (0.84–1.57)	18	25	0.64 (0.34–1.22)	NA
Angermann et al. (2012) [19]	352	363	32	52	0.63 (0.42–0.96)	119	112	1.14 (0.84–1.57)	36	46	0.79 (0.49–1.25)	*SF-36—physical health* STS +2.8; UC +1.3 *p* = 0.03 *SF-36—physical functioning* STS +5.9; UC +1.8 *p* = 0.03
Domingues et al. (2011) [20]	57	63	8	13	0.68 (0.30–1.52)	20	23	0.94 (0.45–1.99)	NA	NA	NA	
Baker et al. (2011) [21]	303	302	0	2	0.20 (0.01–4.13)	NA	NA	NA	NA	NA	NA	*ICICE HFSS* STS +6.7; UC −0.1 *p* < 0.01
Ferrante et al. (2010) [22]	760	758	116	122	0.95 (0.75–1.20)	261	296	0.85 (0.66–1.01)	128	169	0.71 (0.55–0.91)	*MLWHFQ* STS −35.0; UC −30.6 *p* < 0.01
Chaudhry et al. (2010) [23]	826	827	92	94	0.98 (0.75–1.28)	407	392	1.08 (0.89–1.31)	227	223	1.04 (0.84–1.30)	NA
Mortara et al. (2009) [24]	94	160	7	9	1.32 (0.51–3.44)	NA	NA	NA	17	28	1.04 (0.54–2.02)	NA
Bento and Brofman (2009) [25]	20	20	0	1	0.33 (0.01–7.72)	NA	NA	NA	NA	NA	NA	NA
Brandon et al. (2009) [26]	10	10	NA	NA	NA	NA	NA	NA	NA	NA	NA	*MLWHFQ* STS −18.7; UC +6.6 *p* = 0.03
Wakefield et al. (2008) [27]	99	49	25	11	1.12 (0.60–2.09)	41	29	0.49 (0.24–0.98)	51	29	0.73 (0.37–1.46)	*MLWHFQ* STS −16.9; UC −4.0 *p* = NS
Sisk et al. (2006) [28]	203	203	22	22	1.00 (0.57–1.75)	62	74	0.77 (0.51–1.16)	18	29	0.58 (0.31–1.09)	*MLWHFQ* STS 38.6; UC 47.3 Difference −7.3 (−12.1–−2.6) *SF-36 physical health* STS 39.9; UC 36.3 Difference 3.2 (1.0–5.3)
Riegel et al. (2006) [29]	70	65	6	8	0.70 (0.26–1.90)	39	37	0.95 (0.48–1.88)	21	22	0.84 (0.41–1.73)	*MLWHFQ* STS −40.6; UC −43.2 *EQ-5D* STS +0.13; UC +0.12 *PHQ-9* STS −7.3; UC −6.6
DeWalt et al. (2006) [30]	62	65	3	4	0.79 (0.18–3.37)	NA	NA	NA	21	25	0.82 (0.40–1.69)	*MLWHFQ* STS −1; UC −5 *p* = 0.59
Dunagan et al. (2005) [31]	76	75	6	5	1.18 (0.38–3.71)	41	57	0.37 (0.18–0.74)	23	35	0.50 (0.25–0.97)	NA
Cleland et al. (2005) [32]	173	85	27	20	0.66 (0.40–1.11)	85	46	0.82 (0.49–1.38)	34	24	0.62 (0.34–1.14)	NA
Tsuyuki et al. (2004) [33]	140	136	16	12	1.30 (0.64–2.64)	59	51	1.21 (0.75–1.97)	37	38	0.93 (0.55–1.57)	NA
Galbreath et al. (2004) [34]	710	359	54	39	0.70 (0.47–1.04)	NA	NA	NA	NA	NA	NA	*SF-36—general health* STS −0.4; UC +0.2 *p* = 0.87
DeBusk et al. (2004) [35]	228	234	21	29	0.74 (0.44–1.26)	116	117	1.04 (0.72–1.49)	38	43	0.89 (0.55–1.57)	NA
Capomolla et al. (2004) [36]	67	66	5	7	0.70 (0.24–2.11)				17	58	0.05 (0.02–0.12)	NA
Laramee et al. (2003) [37]	141	146	13	15	0.90 (0.44–1.82)	49	46	1.16 (0.71–1.89)	18	21	0.87 (0.44–1.71)	NA
Riegel et al. (2002) [38]	130	228	16	32	0.88 (0.50–1.54)	56	114	0.76 (0.49–1.17)	23	63	0.56 (0.33–0.96)	NA
McDonald et al. (2002) [39]	51	47	3	1	2.76 (0.30–25.67)	NA	NA	NA	1	11	0.07 (0.01–0.53)	NA
Krumholz et al. (2002) [40]	44	44	9	13	0.69 (0.33–1.45)	NA	NA	NA	12	21	0.41 (0.17–1.00)	NA
Kasper et al. (2002) [41]	102	98	7	13	0.52 (0.22–1.24)	NA	NA	NA	26	35	0.62 (0.34–1.13)	*MLWHFQ* STS −28.3; UC −25.7 *p* < 0.01
Jerant et al. (2001) [42]	12	12	1	0	3.00 (0.13–67.06)	NA	NA	NA	1	4	0.18 (0.02–1.95)	NA
Blue et al. (2001) [43]	84	81	25	25	0.96 (0.61–1.53)	NA	NA	NA	12	26	0.35 (0.16–0.76)	NA
Barth (2001) [44]	17	17	0	0	Not estimable	NA	NA	NA	0	0	Not estimable	*MLWHFQ* STS −8.2; UC +0.0 *p* < 0.01
Gattis et al. (1999) [45]	90	91	3	5	0.61 (0.15–2.46)	17	30	0.47 (0.24–0.94)	1	11	0.08 (0.01–0.65)	NA

CI, confidence interval; EQ-5D, EuroQoL 5D; GHQ, general health questionnaire; ICICE HFSS, Improving Chronic Illness care evaluation heart failure symptom scale; MLWHFQ, Minnesota Living with Heart Failure Questionnaire; NA, not available; NS, not significant; OR, odds ratio; PHQ-9, patient health questionnaire-9; SF-36, Short Form survey 36-item; STS, structured telephone support; UC, usual care.

**Table 3:** Baseline characteristics and background HF therapy in studies investigating non-invasive telemonitoring in HF patients.

Author (Year)	Study Acronym	Intervention	Number of Patients	Age	Male (%)	NYHA III/IV (%)	Duration of Follow-Up	Background HF Therapy		
								Beta-Blockers	RAS-Inhibitors	MRAs
Haynes et al. (2020) [47]	BEAT-HF	BP, BW, HR, S and S	288	72 (61–83)	52.7	NA	6 months	NA	NA	NA
Ding et al. (2020) [48]	ITEC-CHF	BW	184	70.1 ± 12.3	76.6	2.1 ± 0.6	6 months	87.5	77.1	57.6
Pekmezaris et al. (2019) [49]		BP, BW, HR, SpO2	104	59.9 ± 15.1	58.7	70.2	3 months	NA	NA	NA
Park et al. (2019) [50]		BP, BW	58	59.1 ± 13.6	67.2	NA	1 month	NA	NA	NA
Koehler et al. (2018) [51]	TIM-HF2	BP, BW, ECG, HR, HS, SpO2	1538	70.0 ± 10.5	69.6	47.5	12 months	91.9	82.5	55.0
Ong et al. (2016) [52]	BEAT-HF	BP, BW, HR, S and S	1437	73.5 (62.5–83.0)	53.8	74.9	6 months	74.7	55.6	19.3
Vuorinen et al. (2014) [53]		BP, BW, S and S	94	58.1 ± 11.8	83.0	61.7	6 months	NA	NA	NA
Villani (2014) et al. [54]	ICARLOS	BP, BW, HR	80	72 ± 3	73.8	3.0 ± 0.5	12 months	NA	NA	NA
Blum and Gotlieb (2014) [55]	MCCD	BP, BW, HR	203	72.5 ± 9.0	71.0	85.5	48 months	68.0	66.0	NA
Seto et al. (2012) [56]		BP, BW, ECG	100	53.7 ± 13.7	79.0	46.0	6 months	98.0	97.0	52.0
Pekmezaris et al. (2012) [57]		BP, Stethoscope	168	82.0 ± 7.0	38.1	20.2	3 months	NA	NA	NA
Lyngå et al. (2012) [58]	WISH	BW	319	73.6 ± 10.1	74.9	100.0	12 months	92.5	96.2	42.6
Dendale et al. (2012) [59]	TEMA-HF 1	BP, BW, HR	160	75.8 ± 9.7	65.0	3.0 ± 0.5	6 months	NA	NA	NA
Wade et al. (2011) [60]		BP, BW	316	76.7 ± 7.0	52.2	NA	6 months	81.6	72.0	NA
Koehler et al. (2011) [61]	TIM-HF	BP, BW, ECG	710	66.9 ± 10.6	81.3	49.9	24 months	92.5	95.4	64.2
Weintraub et al. (2010) [62]	SPAN-CHF II	BP, BW, HR	188	69.0 ± 13.5	66.0	52.7	3 months	88.3	85.6	20.7
Scherr et al. (2009) [63]		BP, BW, HR	108	66 (62–72)	70.8	87.0	6 months	82.4	100.0	40.7
Mortara et al. (2009) [24]	HHH	BP, BW, HR, S and S	461	60 ± 12	85.0	39.9	12 months	87	87	NA
Giordano et al. (2009) [64]		ECG	460	57.0 ± 10.0	85.0	40.4	12 months	72.4	94.3	62.0
Dar et al. (2009) [65]	Home-HF	BP, BW, SpO2, S and S	182	71.0 ± 11,6	66.5	NA	6 months	56.0	87.9	40.7
Woodend et al. (2008) [66]		BP, BW, ECG	121	66.5 ± 12.0	72.0	62.1	12 months	NA	NA	NA
Soran et al. (2008) [67]		BW, S and S	315	76.5 ± 7.0	35.3	41.6	6 months	80.3	97.4	NA
Kashem et al. (2008) [68]		BP, BW, HR, S and S	48	53.5 ± 10.5	74.0	57.5	12 months	NA	NA	NA
Balk et al. (2008) [69]		BP, BW	214	66 (33–87)	70.1	51.9	8 months	80.0	95.3	47.2
Antonicelli et al. (2008) [70]		BP, BW, ECG, HR, UO	57	78.0 ± 7.0	61.4	42.1	12 months	NA	NA	NA
Cleland et al. (2005) [32]	TEN-HMS	BP, BW, ECG, HR	426	67.2 ± 11.6	77.2	34.0	8 months	80.9	81.0	49.1
Goldberg et al. (2003) [71]	WHARF	BW	280	59.1 ± 15.3	67.5	100.0	6 months	37.5	89.6	NA
Benatar et al. (2003) [72]		BP, BW, HR, SpO2	216	63.1 ± 12.9	37.0	3.1 ± 0.3	12 months	53.2	83.8	NA
Jerant et al. (2001) [42]		BW, Stethoscope, S and S	37	70.1 ± 12.1	45.9	35.1	12 months	37.8	67.6	27.0
de Lusignan et al. (2001) [73]		BP, BW, HR	20	75.2	NA	1.8 (1–4)	12 months	NA	NA	NA

BP, blood pressure; BW, body weight, ECG, electrocardiogram; HF, heart failure; HR, heart rate, HS, health status questions; MRA, mineralocorticoid receptor antagonist; NA, not available; NYHA, New York Heart Association; RAS, renin-angiotensin system; SpO2, oxygen saturation; S and S, signs and symptoms; UO, 24 h urine output.

**Table 4:** Clinical outcomes of non-invasive telemonitoring studies.

Author (Year)	Number of Patients		All-Cause Mortality			All-Cause Hospitalization			HF-Related Hospitalization			Quality of Life
			Number of Events		OR (95% CI)	Number of Events		OR (95% CI)	Number of Events		OR (95% CI)	
	TM	UC	TM	UC		TM	UC		TM	UC		
Haynes et al. (2020) [47]	292	NA	NA	NA	NA	NA	NA	NA	NA	NA	NA	NA
Ding et al. (2020) [48]	91	93	2	1	2.07 (0.18–23.21)	73	58	2.45 (1.26–4.76)	15	8	2.10 (0.84–5.22)	*EQ-5D* TM +4.05; UC +1.10 *p* = 0.13
Pekmezaris et al. (2019) [49]	46	58	NA	NA	NA	NA	NA	NA	7	9	0.98 (0.33–2.86)	*MLWHFQ* TM −26.4; UC −32.1 *p* = 0.50 *PHQ-9* TM −2.2; UC −3.0 *p* = 0.43
Park et al. (2019) [50]	60	NA	NA	NA	NA	NA	NA	NA	NA	NA	NA	NA
Koehler et al. (2018) [51]	796	775	61	89	0.64 (0.45–0.90)	262	248	1.04 (0.84–1.29)	NA	NA	NA	*MLWHFQ* TM −3.08; UC −1.98 *p* = 0.26
Ong et al. (2016) [52]	715	722	92	106	0.86 (0.64–1.16)	363	355	1.07 (0.87–1.31)	NA	NA	NA	*MLWHFQ* TM −32.6; UC −28.5 *p* = 0.02
Vuorinen et al. (2014) [53]	47	47	0	0	Not estimable	9	13	0.62 (0.24–1.63)	8	13	0.54 (0.20–1.45)	NA
Villani et al. (2014) [54]	40	40	5	9	0.49 (0.15–1.63)	NA	NA	NA	12	23	0.32 (0.13–0.80)	*PHQ-9* TM −2.8; UC +3.8 *p* < 0.01
Blum and Gotlieb (2014) [55]	104	102	49	45	1.13 (0.65–1.95)	80	74	1.26 (0.67–2.37)	NA	NA	NA	*SF-36—physical health* TM +1; UC +3 *p* = NS *SF-36—mental health* TM +3; UC +6 *p* = NS *MLWFHQ* TM −18; UC −19 *p* = NS
Seto et al. (2012) [56]	50	50	3	0	7.44 (0.37–147.92)	14	10	1.56 (0.61–3.93)	NA	NA	NA	*MLWHFQ* TM −8.9; UC −0.5 *p* = 0.05
Pekmezaris et al. (2012) [57]	83	85	NA	NA	NA	42	41	1.10 (0.60–2.01)	NA	NA	NA	NA
Lyngå et al. (2012) [58]	166	153	5	8	0.56 (0.18–1.76)	79	84	0.75 (0.48–1.16)	NA	NA	NA	NA
Dendale et al. (2012) [59]	80	80	4	14	0.25 (0.08–0.79)	64	66	0.85 (0.38–1.88)	19	34	0.42 (0.21–0.83)	NA
Wade et al. (2011) [60]	164	152	6	6	0.92 (0.29–2.93)	57	49	1.12 (0.70–1.79)	NA	NA	NA	*SF-36—physical health* TM −0.17; UC +1.67 *p* = 0.13 *SF-36—mental health* TM −0.75; UC +0.04 *p* = 0.34
Koehler et al. (2011) [61]	354	356	54	55	0.99 (0.65–1.48)	192	179	1.17 (0.87–1.57)	64	74	0.84 (0.58–1.22)	*SF-36—physical health* TM 54.3; UC 49.9 *p* < 0.05 *PHQ-9* Similar improvement between groups *p* > 0.05
Weintraub et al. (2010) [62]	95	93	1	4	0.24 (0.03–2.16)	29	31	0.88 (0.48–1.62)	10	19	0.46 (0.20–1.05)	NA
Scherr et al. (2009) [63]	66	54	0	1	0.27 (0.01–6.72)	8	17	0.30 (0.12–0.77)	11	17	0.44 (0.18–1.03)	NA
Mortara et al. (2009) [24]	101	160	8	9	1.44 (0.54–3.87)	35	48	1.24 (0.73–2.10)	18	28	1.02 (0.53–1.96)	NA
Giordano et al. (2009) [62]	138	142	11	26	0.39 (0.18–0.82)	67	96	0.57 (0.39–0.84)	43	73	0.49 (0.32–0.76)	NA
Dar et al. (2009) [65]	91	91	6	5	1.21 (0.36–4.13)	23	33	0.59 (0.31–1.12)	10	17	0.54 (0.23–1.25)	*MLWHFQ* No difference between groups *p* = 0.60 *EQ-5D* No difference between groups *p* = 0.50
Woodend et al. (2008) [66]	62	59	5	4	1.21 (0.31–4.73)	60	54	2.78 (0.52–14.91)	NA	NA	NA	*MLWHFQ* No difference between groups *p* = 0.18
Soran et al. (2008) [67]	160	155	11	17	0.60 (0.27–1.32)	75	66	1.19 (0.76–1.86)	30	37	0.74 (0.43–1.27)	*SF-12—physical health* TM 32.3; UC 33.0 *p* = 0.51 *SF-12—mental health* TM 50.2; UC 51.1 *p* = 0.51 *KCCQ—overall summary score* TM 60.2; UC 59.9 *p* = 0.92
Kashem et al. (2008) [68]	24	24	1	1	1.00 (0.06–16.97)	NA	NA	NA	2	10	0.13 (0.02–0.67)	
Balk et al. (2008) [69]	101	113	9	8	1.28 (0.48–3.46)	NA	NA	NA	NA	NA	NA	*Dutch Heart Failure Knowledge Score* No difference between groups *p* = 0.61
Antonicelli et al. (2008) [70]	28	29	3	5	0.58 (0.12–2.68)	9	26	0.05 (0.01–0.23)	NA	NA	NA	*SF-36—health perception* TM + 31; UC + 8 *p* = 0.61
Cleland et al. (2005) [32]	168	85	28	20	0.65 (0.34–1.24)	80	46	0.77 (0.46–1.30)	40	24	0.79 (0.44–1.43)	
Goldberg et al. (2003) [71]	138	142	11	26	0.39 (0.18–0.82)	65	67	1.00 (0.62–1.59)	NA	NA	NA	*SF-36—physical health* TM + 6.7; UC + 4.3 *p* = 0.15 *SF-36—mental health* TM + 5.9; UC + 5.2 *p* = 0.73
Benatar et al. (2003) [72]	108	108	NA	NA	NA	NA	NA	NA	13	24	0.48 (0.23–1.00)	*MLWHFQ* TM − 21.5; UC − 26.3 *p* = 0.98
Jerant et al. (2001) [42]	13	12	0	0	Not estimable	9	12	0.08 (0.00–1.77)	1	4	0.17 (0.02–1.78)	
de Lusignan et al. (2001) [73]	10	10	2	3	0.58 (0.07–4.56)	NA	NA	NA	NA	NA	NA	*GHQ* RM − 4; UC − 7 *p* = NS

CI, confidence interval; EQ-5D, EuroQoL 5D; GHQ, general health questionnaire; KCCQ, Kansas City Cardiomyopathy Questionnaire; MLWHFQ, Minnesota Living with Heart Failure Questionnaire; NA, not available; NS, not significant; OR, odds ratio; PHQ-9, patient health questionnaire-9; SF-12, Short Form survey 12-item; SF-36, Short Form survey 36-item; TM, telemonitoring; UC, usual care.

## Data Availability

Not applicable.
